# NF1 heterozygosity fosters de novo tumorigenesis but impairs malignant transformation

**DOI:** 10.1038/s41467-018-07452-y

**Published:** 2018-11-27

**Authors:** Jean-Philippe Brosseau, Chung-Ping Liao, Yong Wang, Vijay Ramani, Travis Vandergriff, Michelle Lee, Amisha Patel, Kiyoshi Ariizumi, Lu Q. Le

**Affiliations:** 1 Department of Dermatology, University of Texas Southwestern Medical Center, Dallas, TX, 75390 USA; 2 Neurofibromatosis Clinic, University of Texas Southwestern Medical Center, Dallas, TX, 75390 USA; 3 Simmons Comprehensive Cancer Center, University of Texas Southwestern Medical Center, Dallas, TX, 75390 USA; 4 Hamon Center for Regenerative Science and Medicine, University of Texas Southwestern Medical Center, Dallas, TX 75390 USA

## Abstract

Neurofibromatosis type 1 (NF1) is an autosomal genetic disorder. Patients with NF1 are associated with mono-allelic loss of the tumor suppressor gene *NF1* in their germline, which predisposes them to develop a wide array of benign lesions. Intriguingly, recent sequencing efforts revealed that the *NF1* gene is frequently mutated in multiple malignant tumors not typically associated with NF1 patients, suggesting that *NF1* heterozygosity is refractory to at least some cancer types. In two orthogonal mouse models representing NF1- and non-NF1-related tumors, we discover that an *Nf1*^+/−^ microenvironment accelerates the formation of benign tumors but impairs further progression to malignancy. Analysis of benign and malignant tumors commonly associated with NF1 patients, as well as those with high *NF1* gene mutation frequency, reveals an antagonistic role for *NF1* heterozygosity in tumor initiation and malignant transformation and helps to reconciliate the role of the *NF1* gene in both NF1 and non-NF1 patient contexts.

## Abstract (web summary)

Germline mono-allelic loss of the tumour suppressor NF1 predisposes patients to the development of benign lesions but rarely further progression into cancer development. Here the authors use mouse models to show that an NF1 heterozygous microenvironment accelerates the formation of benign tumours but impairs progression to malignancy.

## Introduction

Neurofibromatosis type 1 (NF1) is a tumor predisposition syndrome occurring in 1 in about 3000 births^1^. It is caused by a mutation of the tumor suppressor gene *NF1*, which encodes a negative regulator of the Ras signaling pathway and mainly affects the neural-crest-derived tissue types (e.g. Schwann cells and melanocytes). The phenotype of *NF1* loss-of-function is very broad and encompasses many organ systems at different time points over a lifetime^2^. Indeed, some tumors arising from the nervous system such as plexiform neurofibromas (pNF) can manifest congenitally but occur in about half of all NF1 patients who are examined by whole-body magnetic resonance imaging (MRI) and have a small chance of progression to malignant peripheral nerve sheath tumor (MPNST). Low-grade astrocytomas (mostly optic pathway gliomas) occur in 15–20% of young children with NF1 but are rarely, if ever, congenital. In contrast, some NF1 hallmarks such as cutaneous neurofibromas (cNFs), café au lait macules (CALMs), and iris hamartomas (Lisch nodules) may manifest later in life and be highly penetrant (>99% at age 20).

To explain the high phenotypic variability of NF1, the state-of-the-art working model describes at least three classes of factors^3^. The first factor is the nature of the *NF1* germline mutation. A very small subset of *NF1* mutations can be associated with specific NF1 manifestations in patients^4–7^, indicating that the nature of the mutation may not be a strong determinant. The second factor describes the presence of certain modifiers of neurofibroma that include, but are not limited to, genetic factors^8^, sex hormones^9–13^, and the tumor microenvironment^1,14^. The third factor is related to the inactivation of *NF1* in a specific cell type at a precise moment during a lifetime, the known spatio-temporal loss of *NF1*. A clear example is illustrated by the subtype of patients with the disease manifested in only a fraction of their bodies, hence segmental NF1. The likely explanation is somatic mosaicism, where the first *NF1* mutation occurs later in embryonic development between the blastocyst and the neuroblast stage in such a way that some cells are spared and remain wild type for *NF1*. In some occasions, the disease region may be so discrete that the NF1 diagnostic criteria cannot be met, which would explain the case of a single isolated neurofibroma that can be misinterpreted as a sporadic pNF. Another influential factor may be the nature of the second *NF1* mutation^15^. Thus, our current knowledge and framework to study NF1 is insufficient to predict, even broadly, the prognosis and phenotypic progression of most NF1 patients.

Recently, the advent of next generation sequencing revealed a high mutation frequency for the *NF1* gene in a number of sporadic cancers that are not typically associated with NF1 patients^16^. In some extreme instances, such as desmoplastic melanoma, the *NF1* gene is mutated in up to 90% of the cases. Strikingly, only a single case of desmoplastic melanoma was reported so far in NF1 patients. One possible explanation would be that the *NF1*^+/−^ microenvironment is refractory to malignancy, but this idea contrasts with the established pro-tumorigenic role of the *Nf1*^+/−^ microenvironment in the context of neurofibromagenesis^14^. In fact, NF1 is considered a tumor predisposition syndrome, as NF1 patients have an increased risk of developing multiple neoplasms ^17^. In this paper, we reconcile these discrepancies by compiling experimental and clinical evidence favoring a new framework to study the role of *NF1* in tumorigenesis in both NF1 and non-NF1 contexts. In brief, we discover that an *Nf1*^+/−^ microenvironment accelerates the formation of benign tumors but impairs further progression to malignancy. Analysis of benign and malignant tumors commonly associated with NF1 patients, as well as those with high *NF1* gene mutation frequency, reveals an antagonistic role for *NF1* heterozygosity in tumor initiation and malignant transformation and helps to reconciliate the role of the *NF1* gene in both NF1 and non-NF1 patient contexts.

## Results

### Loss of *Nf1* in keratinocytes does not enhance skin tumors

Despite the characteristic cNF and CALMs, no other skin problem or skin disease is widely associated with NF1 patients even though neurofibromin is expressed in keratinocytes^18–20^. Because the cycles of proliferation and differentiation in keratinocytes occur at a much faster pace than in cells from the nervous system (e.g. Schwann cells), we reasoned that even if a keratinocyte undergoes biallelic *NF1* inactivation, it would be discarded and shed off from the skin rapidly. This would explain why squamous cell carcinoma (SCC) is such a rare event in NF1. Intriguingly, previous studies show indirect evidence suggesting that loss of *Nf1* in keratinocytes may sensitize these cells to tumor development in the context of the two-step carcinogenesis protocol^21^. Briefly, the first step of this protocol consists of applying the mutagenic substance 7,12-dimethylbenz[a]anthracene (DMBA) to induce an activating mutation in the HRAS oncogene, whereas the second step uses a promoting agent such as 12-*O*-tetradecanoylphorbol-13-acetate (TPA) to create an inflammation-rich and tumor-prone microenvironment^22^. Strikingly, *Nf1*^+/−^ mice^23^ (mimicking NF1 patient predisposition mutation) submitted to the two-step carcinogenesis protocol have a much higher rate of papilloma (a precursor lesion to the malignant SCC) development compared to that of the wild-type littermates^21^. This is surprising as mutated HRAS was reported to be unresponsive to RasGAPs such as *Nf1*^24^. Importantly, and in agreement with the virtual absence of phenotypes from keratinocytes of NF1 patients, *Nf1*^+/−^ murine keratinocytes do not spontaneously transform into a tumor in vivo unless additional mutational events are induced^21,23^. Therefore, we reasoned that enhancement of DMBA/TPA-induced tumors in *Nf1*^+/−^ mice was either due to the *Nf1*^+/−^ keratinocytes (independent of its Ras function) or due to an *Nf1*^+/−^ non-keratinocyte cell in the microenvironment.

**Fig. 1:**
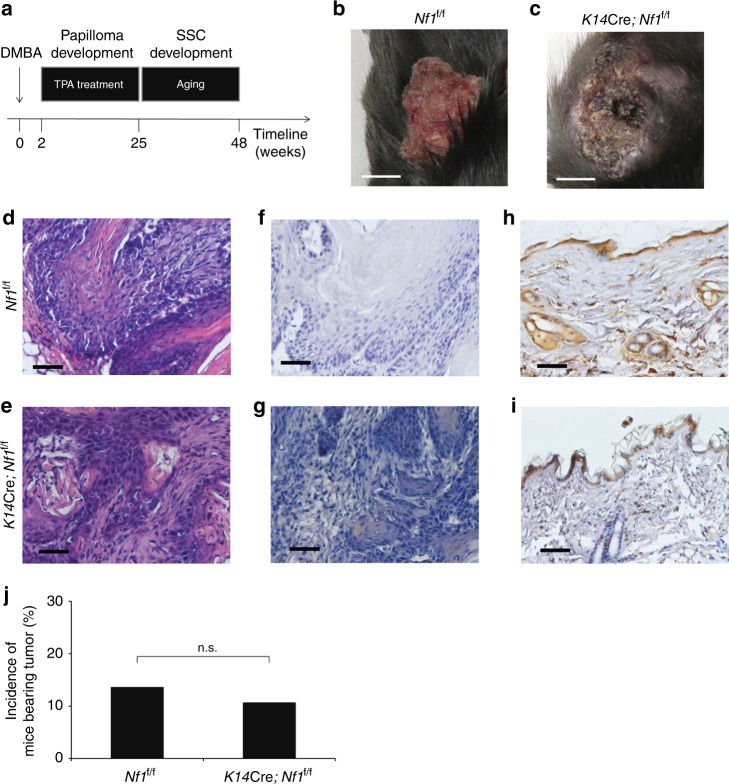
Loss of *Nf1* in keratinocytes does not enhance DMBA/TPA-induced tumors. **a** Schematic of the DMBA/TPA treatment schedule used. Pictures of DMBA/TPA-induced tumors from **b** *Nf1*^f/f^ and **c** *K14*Cre; *Nf1*^f/f^ mice. White scale bar is equal to 5 mm. Representative **d**, **e** H&E-stained and **f**–**i** IHC using K1 antibodies of histological slides of DMBA/TPA-induced tumor from **d**, **f** *Nf1*^f/f^ and **e**, **g** *K14*Cre; *Nf1*^f/f^ and their normal margin from **h** *Nf1*^f/f^ and **i** *K14*Cre; *Nf1*^f/f^. Black scale bar is equal to 50 μm. **j** Tumor incidence in relation to mice genotype. Fischer exact test statistic was performed and is not significant (n.s.). A fraction of *Nf1*^f/f^ (3 out of 22 (14%) and *K14*Cre; *Nf1*^f/f^ (3 out of 28 (11%) developed at least one tumor (papilloma or SCC)

To directly address the first possibility, we decided to develop a new mouse model that would knockout *Nf1* specifically in keratinocytes (*K14*Cre; *Nf1*^f/f^) and challenge it to the two-step carcinogenesis protocol. For this purpose, we crossed the *K14*Cre mice^25^ with the *Nf1*^f/f^ mice^14^ to ultimately obtain *K14*Cre; *Nf1*^f/f^ mice. Next, we submitted 28 *K14*Cre; *Nf1*^f/f^ mice and 22 of their control littermates to the two-step carcinogenesis protocol (Fig. 1a)^22^. After 23 weeks of TPA treatment, *K14*Cre; *Nf1*^f/f^ mice and their control littermates began to develop at least one papilloma. Next, we aged our mice for further tumor development. Interestingly, most tumors evaluated progressed to SCC (Fig. 1b, c). Histological examination by hematoxylin and eosin (H&E) indicates that the majority of the typical papilloma architecture (hyperplastic epidermis surrounding the stroma) progressed to differentiated SCC in tumors from both *Nf1*^f/f^ (Fig. 1d) and *K14*Cre; *Nf1*^f/f^ (Fig. 1e) mice. In addition, immunohistochemistry using keratin 1 (K1, an epithelial cell marker that is lost upon cancer progression^22^) antibodies confirmed the low expression of K1 in tumor samples from both *Nf1*^f/f^ (Fig. 1f) and *K14*Cre; *Nf1*^f/f^ (Fig. 1g) mice, although K1 is highly expressed in normal margin epidermis (Fig. 1h, i). Overall, we could not discriminate mice genotypes based on histology. Of note, there was no statistical difference (exact Fischer test value = 1) between the number of mice bearing a tumor in the *Nf1*^f/f^ group (14%) vs the *K14*Cre; *Nf1*^f/f^ group (11%) (Fig. 1j). These results indicated that *Nf1* biallelic inactivation in keratinocytes does not enhance DMBA/TPA-induced malignant tumors and ruled out the possibility that the sensitization phenotype initially observed by Atit et al.^21^ may be due to some Ras-independent function of *Nf1* in keratinocytes. Consistently, Atit et al.^21^ was not able to demonstrate the *Nf1* loss of heterozygosity or the loss of *Nf1* expression in keratinocytes of their papillomas. Hence, we reasoned that the sensitization to tumor development observed by Atit et al.^21^ in the *Nf1*^+/−^ mice was the consequence of *Nf1* gene dosage in one or more non-keratinocyte cell (a cell from the microenvironment).

**Fig. 2:**
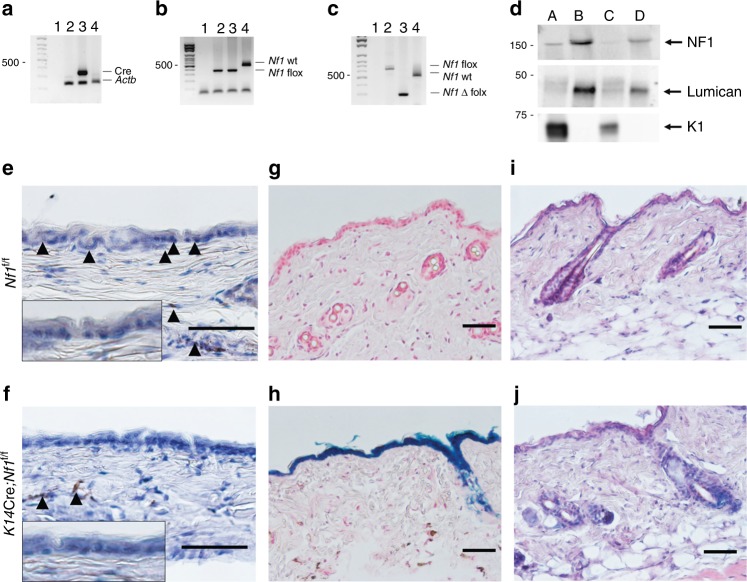
Loss of *Nf1* in keratinocytes does not trigger obvious skin phenotype. Mice genotyping with specific PCR primers for **a** Cre recombinase, **b** *Nf1* flox allele and **c** *Nf1* deleted. Lane 1 = no template control, lane 2 = DNA from *Nf1*^f/f^ mice’s skin tail, lane 3 = DNA from *K14*Cre; *Nf1*^f/f^ mice’s skin tail, lane 4 = DNA from wild-type mice’s skin tail. **d** Western blot of skin protein extract quantified using anti-NF1, anti-LUMICAN (dermal marker), and anti-K1 (epidermal marker). Lane A = epidermis from *Nf1*^f/f^ mice; Lane B = dermis from *Nf1*^f/f^ mice; Lane C = epidermis from *K14*Cre; *Nf1*^f/f^ mice; Lane D = dermis from *K14*Cre; *Nf1*^f/f^ mice. **e,** **f** Representative IHC using anti-NF1 antibodies of dissected normal dorsal skin from **e** *Nf1*^f/f^ and **f** *K14*Cre; *Nf1*^f/f^ (arrow head pointing to NF1 expression in brown). **g,** **h** Representative X-gal staining counterstained with Nuclear Fast Red of dissected normal dorsal skin from **g** *Nf1*^f/f^; *ROSA-LacZ* and **h** *K14*Cre; *Nf1*^f/f^; *ROSA-LacZ* mice. **i,** **j** Representative H&E of dissected normal dorsal skin from **i** *Nf1*^f/f^ and **j** *K14*Cre; *Nf1*^f/f^. Scale bar is equal to 50 μm

To further validate the *K14*Cre; *Nf1*^f/f^ mouse model, expected genotypes were confirmed by PCR (Fig. 2a, b). We also evaluated the extent of Cre recombinase action to delete NF1 in the epidermis by four means. First, we analyzed the *Nf1-*deleted band from mice’s skin tail by PCR^26^. Indeed, the *Nf1*-deleted band is virtually absent from *K14*Cre; *Nf1*^f/f^ mice, whereas the band is still present in skin from *Nf1*^f/f^ mice (Fig. 2c). Second, we prepared protein extract from purified epidermis and dermis from *K14*Cre; *Nf1*^f/f^ and *Nf1*^f/f^ mice and analyzed the extent of NF1 knockout by western-blotting (Fig. 2d). Third, we performed immunohistochemistry in skin from *K14*Cre; *Nf1*^f/f^ and *Nf1*^f/f^ mice using anti-NF1 antibodies (Fig. 2e, f). Fourth, we crossed the *K14*Cre; *Nf1*^f/f^ mice with the *ROSA*-LacZ reporter mouse and observed specific signal in the epidermis upon X-Gal staining (Fig. 2g, h). Overall, we did not identify any skin phenotype in *K14*Cre; *Nf1*^f/f^ mice (Fig. 2i, j).

### Dual role of *Nf1*^+/−^ microenvironment in skin tumorigenesis

**Fig. 3:**
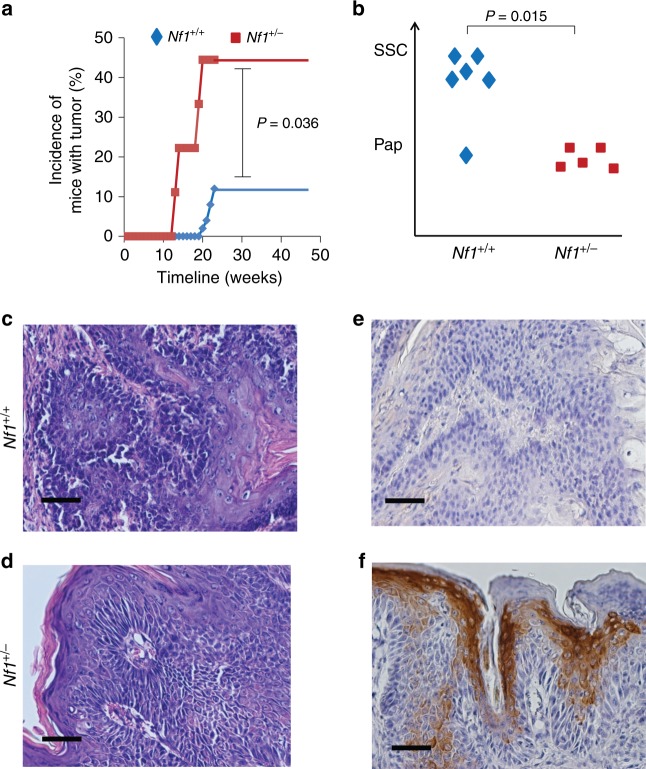
Antagonistic role of *Nf1*^+/−^ microenvironment in DMBA/TPA-induced tumors. **a** Incidence of mice developing tumor under DMBA/TPA treatment as a function of time. A fraction of mice with *Nf1*^+/+^ (6 out of 50; 12%) and *Nf1*^+/−^ (4 out of 9; 44%) microenvironment developed at least one tumor (papilloma or SCC). Fischer exact test statistic was performed. **b** Tumors from six mice with *Nf1*^+/+^ and five *Nf1*^+/−^microenvironment were reviewed by a pathologist and classified as papilloma (benign) or SCC (malignant) based on **c**–**f**. Fischer exact test statistic was performed. Representative **c,** **d** H&E-stained and **e,** **f** IHC using K1 antibodies in histological sections of dissected DMBA/TPA-treated dorsal skin from **c**, **e** *Nf1*^+/+^ and **d**, **f** *Nf1*^+/−^. Scale bar is equal to 50 μm

To determine the contribution of an *Nf1*^+/−^ microenvironment to the development of papillomas, additional mice were submitted to the two-step carcinogenesis protocol as described in Fig. 1a. Papilloma development was assessed each week by visual inspection of the dorsal skin. Strikingly, a large fraction of *Nf1*^+/−^ mice (44%, 4 out of 9) developed papilloma, whereas no (0 out of 50) *Nf1*^f/f^ (wild-type *Nf1* in all tissues) or *K14*Cre; *Nf1*^f/f^ (*Nf1* knockout in keratinocytes but wild type in all other tissues) mice, here after called *Nf1*^+/+^ mice (all *Nf1*^+/+^ mice are from Fig. 1 in the previous section), showed any sign of papilloma formation at week 19 (Fig. 3a). Eventually, some *Nf1*^+/+^ mice developed papilloma but to a much lesser extent (12%, 6 out of 50; Fischer exact test value = 0.036). This indicates that an *Nf1*^+/−^ microenvironment accelerate papilloma formation.

To determine the influence of an *Nf1*^+/−^ microenvironment on the progression from papillomas to SCC, we further aged the mice that developed papillomas from both genotypes (*Nf1*^+/+^ and *Nf1*^+/−^) for an additional period of 23 weeks. Strikingly, in the following months after TPA treatment, the gross morphology of all the papillomas evaluated from the *Nf1*^+/−^ mouse model did not progress further and remained benign. However, most of the tumors from the *Nf1*^+/+^ mice did progress to frank malignant carcinoma lesions (Fischer exact test value = 0.015; Fig. 3b). Histological examination by H&E confirmed that most tumors from the *Nf1*^+/+^ mice progressed to differentiated SCC (Fig. 3c), whereas all tumors from *Nf1*^+/−^ mice maintained their typical papilloma architecture (Fig. 3d). In addition, immunohistochemistry using the epithelial marker K1 confirmed its expression only in tumors from *Nf1*^+/−^ mice (Fig. 3e, f). This indicated that an *Nf1*^+/−^ microenvironment repressed the progression from the benign papilloma to SCC. Altogether, these results illustrate the antagonistic role of the *Nf1*^+/−^ microenvironment regarding cancer progression, in which it fosters de novo benign tumorigenesis but impairs malignant transformation.

### Dual role of *Nf1*^+/−^ microenvironment in the clinical context

In the context of NF1, patients are by definition heterozygous for the *NF1* gene due to inactivation of one copy of *NF1* in all their somatic cells, but these patients rarely develop papillomas or SCC. Therefore, we extensively and systematically searched the literature for clinical data on NF1-associated benign and malignant tumors (Supplementary Table 1). First, we noticed that the NF1-associated neoplasms have an early onset (Supplementary Table 1). Indeed, the penetrance is close to 100% by age 20 for cNFs and iris hamartomas that are characteristic of NF1 patients. In fact, the great majority of benign lesions in NF1 patients develop with much less frequency in the general population. In other words, the development of these tumors and hamartomas seems to be promoted by an *Nf1*^+/−^ microenvironment. Thus, our initial observation from the pre-clinical model representing a non-NF1-associated tumor (i.e. the DMBA/TPA-induced tumor) is mirrored clinically in the context of NF1.

Second, most NF1-associated neoplasms are of the benign type and rarely, if ever, progress to malignant tumors (Supplementary Table 1). This is especially true for the tumors characteristic of NF1 patients. For the group of NF1-related tumors with much lower penetrance: low-grade pilocytic astrocytomas, low-grade gastrointestinal stromal tumors (GISTs), and most pheochromocytomas are rarely malignant in people with NF1. In fact, the prognosis is actually better for NF1 patients compared to non-NF1 patients for low-grade pilocytic astrocytomas and GISTs (Supplementary Table 1).

Third, we noticed that sporadic cancer with high mutation frequency for the *NF1* gene (e.g. melanoma, glioblastoma, lung, skin, and ovarian cancer), rarely, if ever, develop in NF1 patients, although they have already lost one copy of *NF1* (Supplementary Table 1; Supplementary Table 2). This argues in favor of a model where the *NF1*^+/−^ microenvironment impairs certain carcinogenesis. Altogether, these human clinical data align well with our conclusion derived from the DMBA/TPA-induced tumor study (Fig. 3), in which an *Nf1*^+/−^ microenvironment played an antagonistic role throughout the stepwise cancer progression.

### A new mouse model of progression from neurofibroma to MPNST

**Fig. 4:**
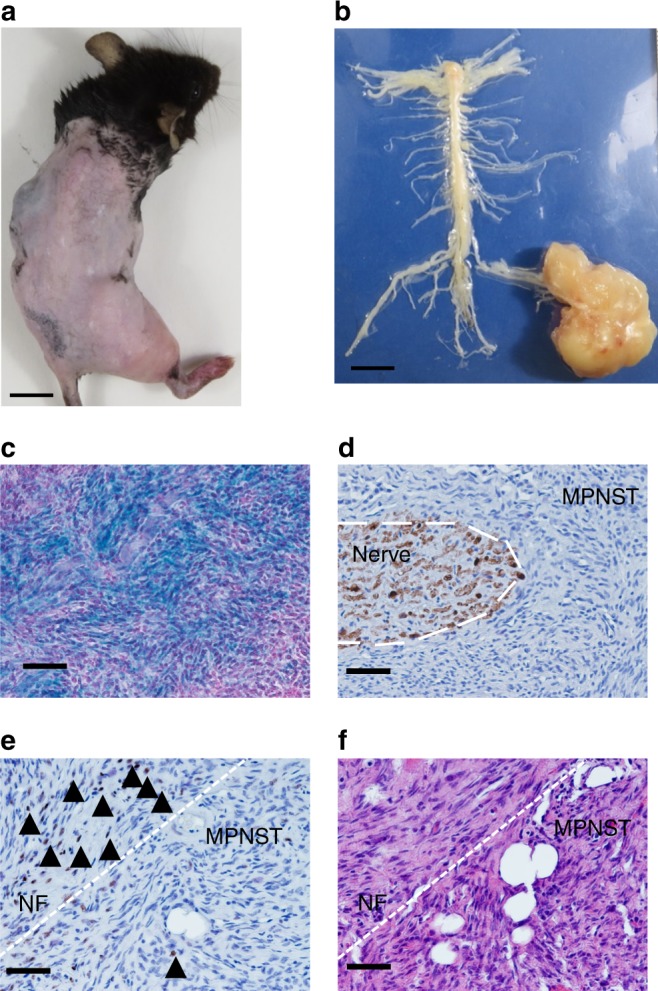
A novel mouse model to study the sequential progression from plexiform neurofibroma to MPNST. **a**, **b** Representative gross image of **a** *PLP*CreERT2; *Nf1*^f/f^ mouse that develops MPNST in the right sciatic nerve and **b** its dissected spinal cord along with peripheral nerves. **c**–**f** Representative **c** X-Gal staining (*PLP*CreERT2; *Nf1*^f/f^; *ROSA*-LacZ), IHC using **d** S100 and **e** H3K27 (arrow head) antibodies and **f** H&E in histological sections of dissected MPNST from *PLP*CreERT2; *Nf1*^f/f^. Scale bar is equal to 50 μm

An apparent exception to this rule seems to be MPNST, which broadly refers to a collection of malignant sarcomas with connection to the nerve structure. MPNST can develop sporadically in non-NF1 patients, as a consequence of radiation therapy, or in the context of NF1. Little is known about the contribution of the *Nf1*^+/−^ microenvironment on the progression from pNF (benign) to MPNST (malignant). This is due in part to the lack of an animal model describing the transition from pNF to MPNST^27–29^. Myelin-specific proteolipid protein (PLP) is expressed in myelinating Schwann cells from precursor stages and lasts throughout their mature stages^30^. In a *PLP-CreERT2* pNF model, we have previously demonstrated that Schwann cells are susceptible to pNF development when *Nf1* is ablated with tamoxifen induction at the immature Schwann cell stage (new born)^31^. In the course of a study on the role of the stem cell factor in pNF development using the *PLP*CreERT2; *Nf1*^f/f^ mice^32^, our lab observed that some pNF further transformed into MPNST spontaneously (Fig. 4a, b). These sarcomas developed with connection to the nerve structure (Fig. 4b). To make sure that these tumors originated from *Nf1*^−/−^ Schwann cells, we crossed the *PLP*CreERT2; *Nf1*^f/f^ mice to *ROSA*-LacZ reporter mice and observed a specific signal from cancer cells upon X-Gal staining (Fig. 4c). As opposed to pNF, MPNST often present the loss of a Schwann cell differentiation marker such as S100 (Fig. 4d) as well as the epigenetic repressive marker H3K27Me3 (Fig. 4e). Of note, some tumors show a mixture of neurofibroma histology and MPNST histology with much higher cellularity (Fig. 4f), mirroring initial observations on human clinical samples^33^. Thus, the *PLP*CreERT2; *Nf1*^f/f^ mice are an excellent model to decipher the role of the *Nf1*^+/−^ microenvironment in the context of the spontaneous progression from pNF to MPNST.

### Dual role of *Nf1*^+/−^ microenvironment in NF1-associated tumor

**Fig. 5:**
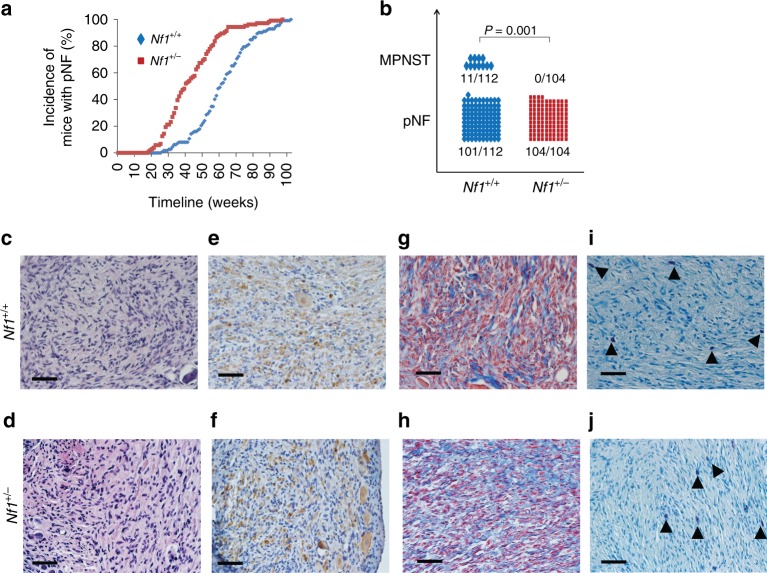
Antagonistic role of *Nf1*^+/−^ microenvironment in plexiform neurofibroma and MPNST formation. **a** Incidence of mice with plexiform neurofibroma (pNF). **b** Tumor were classified as pNF (benign) or MPNST (malignant) based on histology. Fischer exact test statistic was performed. **c**–**j** Representative **c**, **d** H&E; **e**, **f** IHC using S100 **g,** **h** trichrome and **i**, **j** toluidine blue (arrow head pointing toward mast cells) staining in histological sections of dissected pNF from *PLP*CreERT2; *Nf1*^f/f^ (**c**, **e**, **g**, **i**) and *PLP*CreERT2; *Nf1*^f/−^ (**d**, **f**, **h**, **j**). Scale bar is equal to 50 μm

To evaluate the contribution of the *Nf1*^+/−^ microenvironment to cancer progression in a mouse model that develops NF1-associated benign and malignant tumors, we analyzed a cohort of 216 *PLP*CreERT2; *Nf1*^f/f^ (*Nf1* knockout in Schwann cells, but wild type in all other tissues) and *PLP*CreERT2; *Nf1*^f/−^ (*Nf1* knockout in Schwann cells, but heterozygous in all other tissues) mice. Although we could observe high penetrance of pNF even in *PLP*CreERT2; *Nf1*^f/f^ mice, pNF develops much faster in the *PLP*CreERT2; *Nf1*^f/−^ (Fig. 5a). At one year of age, the majority of *PLP*CreERT2; *Nf1*^f/−^ (77 out of 104, 74%) mice already developed pNF and showed the clinical signs of pNF (scruffy fur, limping, paralysis), whereas only a minority of *PLP*CreERT2; *Nf1*^f/f^ (32 out of 112, 29%) mice did (Fig. 5a). Next, mice that developed pNF were further aged to evaluate their capacities to progress to MPNST. Strikingly, no MPNST developed in *PLP*CreERT2; *Nf1*^f/^^−^ mice (0 out of 104, 0%), whereas all MPNST observed arose from *PLP*CreERT2; *Nf1*^f/f^ mice (10%, 11 out of 112) (exact Fischer test value = 0.001) (Fig. 5b). It is likely that given more time, some *PLP*CreERT2; *Nf1*^f/−^ mice will develop MPNST as in patients with NF1. However, it is clear that at the time of sacrification secondary to morbidity from the pNF, we only observed MPNST development in *PLP*CreERT2; *Nf1*^f/f^ mice. This indicates that in the context of NF1-associated tumors, the *Nf1*^+/−^ microenvironment has the capacity to accelerate the formation of benign lesions (pNF) but restrain the further progression to frank sarcoma (MPNST), as observed with models of non-NF1-associated papilloma and SCC (i.e. DMBA/TPA-induced skin tumors).

Our results suggest a novel framework to study the role of *NF1* in tumorigenesis and predict that identifying the cells and factors responsible for slowing down malignant progression may be of significance to NF1 patients. To get mechanistic insights about the cells involved, we proceed with a systematic histological evaluation highlighting the key hallmarks of pNF (Fig. 5c–j). We found no obvious difference by H&E (Fig. 5c, d), S100 immunohistochemistry (Schwann cells) (Fig. 5e, f), trichrome staining (collagen) (Fig. 5g, h), and toluidine blue (mast cells) (Fig. 5i, j) when we compared pNF from *PLP*CreERT2; *Nf1*^f/f^ and *PLP*CreERT2; *Nf1*^f/−^ mice. In fact, human cNF and pNF have almost identical histology although some pNF further progress to MPNST but not cNF. Therefore, we reasoned that the cell types slowing down malignant progression may be driven by a minor population of cells not obviously picked up by routine histological evaluation.

### *Nf1*^+/−^ background enhances T cell proliferation and function

**Fig. 6:**
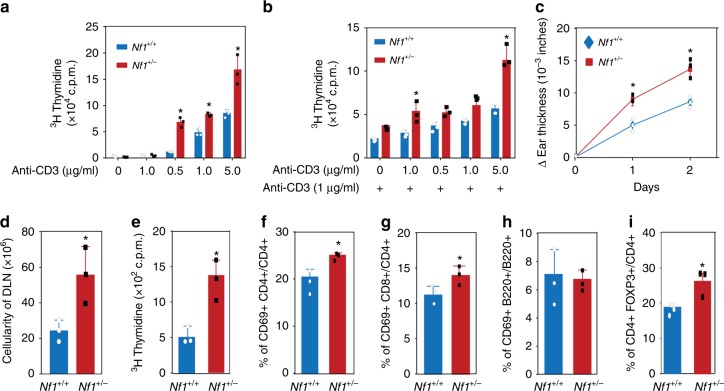
Impact of *Nf1* heterozygosity on T cells proliferation and function. **a**, **b** Proliferation of peripheral T cells from *Nf1*^+/+^ and *Nf1*^+/−^ mice stimulated by various concentrations of **a** anti-CD3e antibody alone or with **b** CD28 costimulation. Proliferation was assessed in biological duplicate and technical triplicate by ^3^H-thymidine incorporation during the last 20 h of culture. **c** Delayed-type hypersensitivity (DTH) assay: On day 0, *Nf1*^+/+^ and *Nf1*^+/−^ mice were sensitized on abdominal skin by topical application of oxazolone. On day 6, *Nf1*^+/+^ and *Nf1*^+/−^ mice were challenged by topical application on the left and right ear with oxazolone and control solvent, respectively. DTH was assessed daily through by measuring ear thickness (Δ). Experiments were performed in biological triplicates. **d** Two days after challenge, cellularity of draining lymph nodes (DLNs) of *Nf1*^+/+^ and *Nf1*^+/−^ mice were determined. **e** Spontaneous activation of cells from DLNs (T cells) was measured by ^3^H-thymidine incorporation after culturing for 3 days without stimuli. **f** Fraction of CD4^+^ cells that are activated. **g** Fraction of CD8^+^ cells that are activated. **h** Fraction of B cells^+^ that are activated. **i** Fraction of Treg^+^ cells in total CD4^+^ subset. Panels **d**–**i** were performed in biological triplicates. Statistical paired *t*-test performed; * *P*< 0.05. Variation was estimated using standard deviation with a 95% confidence level

Although largely unexplored, there is some evidence in the NF1 literature suggesting that NF1 patients have an enhanced immunosurveillance system that could impair malignant transformation^34,35^. Therefore, we further turned our attention to the immune system in *Nf1*^+/−^ mice. We isolated T cells from lymph nodes and submitted them to an in vitro T cell proliferation assay, an established and classic method to determine the reactivity and functional status of the T lymphocytes^36^. We found that *Nf1*^+/−^ T cells are more hyperproliferative when stimulated with anti-CD3 than their wild-type counterparts (Fig. 6a, b). Most importantly, to evaluate the functional responses of T cells to antigens in vivo, we utilized the delayed-type hypersensitivity (DTH) assay, a classic method for evaluating T cell-mediated immune responses^37,38^. The DTH reaction is divided into the sensitization and elicitation phases. During the initial phase of this model, the ears from *Nf1*^+/−^ or *Nf1*^+/+^ mice are immunized by topical application of oxazolone antigen. The second phase is initiated 5–12 days after sensitization, whereby the previously sensitized mice are challenged by topical application of the same antigen at the ears. The DTH response is evaluated 24 h post-challenge by measuring the ear thickness. The skin thickness, which is a surrogate for a massive immune cell infiltration and enhanced immunity, was more pronounced in *Nf1*^+/−^ mice (Fig. 6c). There was also much higher cellularity and the T cells were more hyperproliferative in the draining lymph nodes of *Nf1*^+/−^ mice (Fig. 6d, e). Detailed FACS analysis indicates again that the number of activated T cells, including the fraction of activated cytotoxic CD8+ T cells, is higher in the draining lymph nodes of *Nf1*^+/−^ mice compared to *Nf1*^+/+^ mice (Fig. 6f–i). Altogether, this indicates that the *Nf1*^+/−^ background enhances the immunity by increasing the number of hyperproliferative *Nf1*^+/−^ T cells and augmenting the functional responses of T cells, the major contributors to immune surveillance responsible to clear out malignant cells, to antigens in vivo.

## Discussion

**Fig. 7:**
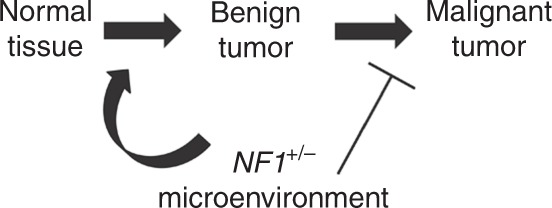
*NF1* heterozygosity fosters de novo tumorigenesis but impairs malignant transformation. Schematic summarizing the antagonistic role of *NF1*^+/−^ microenvironment on the sequential cancer progression from normal to benign to malignant tumor

The goal of the present report is to propose a novel framework to study the role of *NF1* in tumorigenesis that integrates a number of apparent discrepancies in the published NF1 literature together with the current reported data. On the one hand, our model stipulates that an *Nf1*^+/−^ microenvironment accelerates the formation of benign tumors (Fig. 7). Using two independent mouse models, we observed a faster appearance of benign tumors in the context of NF1- (Fig. 5a) and non-NF1- (Fig. 3a) associated tumors. This is in agreement with the findings of the Parada^14^ and Gutmann^39^ labs in the context of pNF and astrocytoma (low-grade optic glioma), respectively, in which they concluded that an *Nf1*^+/−^ microenvironment is critical for these tumors’ formation. Their results help explain why NF1 patients are predisposed to these benign tumors and why sporadic pNFs are a rare event. However, other investigators described mouse models where neurofibromas are much less dependent on the *Nf1*^+/−^ microenvironment^40–42^. This discrepancy may be due to different pools of neurofibroma cells of origin targeted by different Schwann cell Cre lines. Importantly, in all mouse models reported so far, including non-NF1-related tumor models, there was either no impact or a positive impact toward formation of benign tumors. Thus, this suggests that in both humans and mice the *Nf1*^+/−^ microenvironment favors the development of benign tumors.

On the other hand, our model predicts that an *Nf1*^+/−^ microenvironment prevents further progression to frank malignant lesions (Fig. 7). At first glance, this statement may seem paradoxical to the established cancer predisposition feature of the *NF1* gene. It is true that NF1 patients have a higher lifetime risk of developing a cancer compared to the general population^17^. However, as summarized in Supplementary Table 1, the great majority of the tumors found in NF1 patients will rarely, if ever, progress to an aggressive malignant tumor. Indeed, the malignant state of a tumor is not used as a diagnostic criteria for NF1^2^. Although NF1 patients have a 10–15 years shorter life expectancy, the mean age at diagnosis for most tumors with high *NF1* mutation frequency falls within their lifetime (Supplementary Tables 1 and 2), ruling out the arguments that NF1 patients would not live long enough to actually develop frank cancers.

Since NF1 patients have a 10% lifetime chance of being ultimately diagnosed with MPNST and the incidence of MPNST is very low in the general population, this leads to the general idea that NF1 patients, harboring an *NF1*^+/−^ microenvironment, are more susceptible to this cancer development. In support of this theory, NF1 patients have a worse prognosis compared to that of sporadic MPNST cases. However, due to its deeper anatomical location, NF1-associated MPNST is typically diagnosed at a later stage compared to non-NF1 patients, which could explain the poorer prognosis of NF1 patients with MPNST (and not necessarily because of the *NF1* status of the microenvironment)^43–45^.

The consistent experimental and clinical evidence present in this study predicts that the number of MPNST cases in NF1 patients is actually much lower due to the antagonistic role of the *NF1*^+/−^ microenvironment, considering the size and number of pNF in NF1 patients. Indeed, one of the major confounding factors in the current interpretation of death certificates and cohort studies resides in the lack of data describing precursor lesions of MPNST. It is conceivable that a population with an increased chance of developing benign lesions (i.e. NF1 patients with pNF) is more prone to develop MPNST than the general population (i.e. non-NF1 patients without pNF). To delineate the contribution of *NF1* heterozygosity to malignancy, one would have to evaluate the percentage of non-NF1 patients with pNF that ultimately develop MPNST. Unfortunately, most, if not all, MPNST (either from NF1 or non-NF1 patients) are rarely diagnosed at the level of the precursor pNF lesion due to their deep anatomical location, precluding anyone from conclusively answering this critical question. In other words, our model predicts that the incidence of MPNST, and breast cancer^46–50^ as well as juvenile myelomonocytic leukemia (JMML)^51–53^ that are associated with NF1, would be much higher in NF1 patients if it were not due to the repressive role of the *NF1*^+/−^ microenvironment.

Some other NF1-associated tumors worth mentioning (e.g. gliomas, GISTs, pheochromocytomas) may have been referred to as malignant in the literature. Generally, they are not malignant, although some cases of high-grade astrocytoma, malignant GIST, and malignant pheochromocytoma were reported in NF1 patients (Supplementary Table 1) and have a more benign course compared to their sporadic counterpart. This is reflected in their better prognosis (Supplementary Table 1).

Our results suggest a novel framework to study the role of *NF1* in tumorigenesis (Fig. 7). Importantly, it predicts that identifying the cells and factors responsible for (1) accelerating benign tumor formation and (2) slowing down malignant progression may be of significance to NF1 patients. Regarding the former mechanism, it is interesting to note that drastically reducing the number of mast cells abrogated the neurofibromatogenesis accelerating capacity of the *Nf1*^+/−^ microenvironment in our PLPcreERT2 mouse model^32^. We conclude that mast cells are not absolutely required for neurofibroma but will make neurofibroma grow faster in this model. The targeting of mast cells is not unfamiliar to the NF1 field. Since mast cells are known to secrete histamine, which is largely involved in allergic reactions, mast cells have been first hypothesized as responsible for skin pruritus^54^. Still, today there is no conclusive evidence regarding the cause of pruritus in the context of cNF. In a series of landmark papers^55–57^, the positive correlation of mast cell depletion and loss of the acceleration of pNF formation, coupled to the drastic effect of a mast cell inhibitor in an NF1 patient with morbid pNF, stimulated the Clapp group to launch a clinical trial targeting advanced pNF. Disappointingly, the great majority of the patients did not respond to the mast cell inhibitor treatment^58^. Our new framework predicts that mast cell inhibitors may be more effective towards early tumor development as a mean of delaying tumor onset rather than targeting mechanisms of neurofibroma maintenance.

Recently, independent labs have reported macrophages as an emerging hallmark of neurofibroma^32,59^. Macrophage density correlates with disease progression^59^, suggesting a pro-tumorigenesis role for macrophages. Pharmacological inhibition of macrophage proliferation correlates with neurofibroma volume reduction in vivo^59^. However, the compound used is a non-specific dual mast cell and macrophage inhibitor, precluding any definitive conclusion on the functional role of macrophages in vivo. In the context of optic pathway glioma, another NF1-associated benign tumor, *Nf1*^+/−^ microglia (macrophage-like cells) enable tumor growth^60^. Thus, *Nf1*^+/−^ macrophages in the tumor microenvironment have an established pro-tumorigenesis role and interfering with macrophage function may be of therapeutic interest for NF1 patients.

The field of cancer immunology holds great promises in leveraging the natural capacity of immune surveillance to fight against cancer. Indeed, there is indirect evidence from a case report of NF1 patients undergoing organ transplants that suggests that immune surveillance in NF1 patients suppress malignant transformation^35^. Here, we discovered that *Nf1*^+/−^ T cells are hyperproliferative and have an enhanced immunity signature, including expanding the fraction of activated cytotoxic CD8+ T cells. This suggests that an *Nf1*^+/−^ microenvironment may empower NF1 patients with an enhanced immune surveillance system and may explain why most NF1 patients do not develop MPNST. Indeed, in NF1 patients, a negative correlation between the number of activated T cells and the progression from pNF to MPNST in NF1 patients was recently reported^34^. Importantly, this mechanism is not perfectly effective, as some NF1 patients do develop malignant tumors. Therefore, we reasoned that if the putative enhanced immune surveillance capacity of NF1 patients is a major force contributing to the malignant progression impairment, then it will be proportionally effective in highly immunogenic tumors. Indeed, the few malignant tumors associated with NF1 patients, such as MPNST and breast cancer, have a low immunogenic potential^61–63^, and the cancer types with high mutation frequency for the *NF1* gene that are not associated with NF1 patients, such as skin, lung, ovarian, melanoma, and bladder cancers, are highly immunogenic^64,65^. Altogether, we propose that the enhanced function of some *NF1*^+/−^ immune cells in the tumor microenvironment of NF1 patients may favor the benign tumor state (i.e. *NF1*^+/−^ mast cells, macrophages), but can also impair malignant transformation (i.e. *NF1*^+/−^ T cells).

In summary, we present consistent evidence in both human and mouse models that the *NF1*^+/−^ microenvironment has an antagonistic role in tumorigenesis: accelerating development of benign tumors and restraining further progression to malignant cancer. Our novel framework to study the role of *NF1* in tumorigenesis integrates the concept of *NF1* as a tumor predisposition gene, knowledge derived from the Krox20-Cre; *Nf1*^*f*/−^ mouse model^14,66^, while at the same time explaining why NF1 patients develop mostly benign tumors instead of developing the cancer types with high *NF1* mutation rates as seen in the general population. Further elucidating the mechanisms underlying the impairment of cancer progression by tumor microenvironment cells harboring a tumor suppressor genes in the haploinsufficient state, such as *NF1*^+/−^, may translate into novel therapeutic strategies.

## Methods

### Animal studies

All mice were housed in the Animal Care Facility at the University of Texas Southwestern Medical Center, and all procedures were approved by Institutional Animal Care and Use Committee (IACUC) at the University of Texas Southwestern Medical Center and conformed to NIH guidelines. All mice strains were maintained in a room with 12/12 (day/night) light cycle with a temperature of 70–72 °F. *K14*Cre mice were available from the Jackson Laboratory (Bar Harbor, ME). All mice were maintained on a mix C57bl/6 background. DMBA/TPA treatment was performed according to Abel et al.^22^. using a single dose of DMBA (25 μg) and a bi-weekly schedule for TPA (4 μg) over 23 weeks. The *PLP*CreERT2; *Nf1*^f/−^; *ROSA*-LacZ mice^31^ was conditionally activated with a single subcutaneous dose of 4-hydroxytamoxifen (40 μg) dissolved in 100% ethanol on the first postnatal day. Genotyping was performed by PCR as reported elsewhere^14,23,26,67^. A total number of 59 mice (DMBA/TPA study) and 216 mice (MPNST study) were used in this report. For the DMBA/TPA study, full randomization was not possible to maximize the number of same sex mice and enrolled on treatment or vehicle at 3 months old. The investigators were not blinded to the group allocation during the experiment. No mice were excluded for any reasons.

### Histology and Immunohistochemistry

Tissues were fixed in 10% formalin-buffered solution for at least 48 h. Then tissues were paraffin embedded (Rushabh Instruments LLC), were sectioned at 5 μm using a microtome (Leica RM2135), and were allowed to dry on superfrost glass slide at room temperature (r.t.). H&E staining was performed using Gill 3 hematoxylen (Thermo Scientific, 72604), followed by short washes with High Def (Statlab, SL103) and Bluing reagent (Fischer 22-220-106) and eosin-Y with phaloxine (Thermo Scientific, 71304) as counterstaining. For X-Gal staining, tissue were initially incubated in 4% para-formaldehyde (10 min, r.t.), were rinsed twice with phosphate-buffered saline (PBS) 1× followed by incubation into X-Gal staining solution (4-chloro-5-bromo-3-indolyl-b-galactoside (X-Gal) (1 mg/ml), potassium ferrocyanide (4 mM), potassium ferricyanide (4 mM), MgCl_2_ (2 mM) in PBS) for 24–48 h before fixation by 10% formalin-buffered solution. Tissue slides were counterstained with Nuclear Fast Red (Millipore, HC609588) for 5 min. For immunohistochemistry, the Vecta Stain Elite ABC kit (Vectorlabs, PK-6100) was used according to the manufacturer’s protocol. The following primary antibodies were used in the immunohistochemistry studies: K1 (BioLegend, 905601, 1:100), NF1 (Santa Cruz; sc-67, 1:100), H3K27Me3 (Cell Signaling, 9733 S, 1:500), and S100 (DAKO, Z0311, 1:5000). All histologies were repeated in at least three biological replicates. The pathologist (T.V.) was blinded to the mice genotype when performing the histological review.

### Western blot

Epidermis was enzymatically digested with dispase and physically separated from dermis using tweezers. Purified cells were lyzed and analyzed by immunoblot analysis with the following antibodies: NF1 (Santa Cruz; H-12, 1:100); K1 (BioLegend, 905601, 1:1000); LUMICAN (Santa Cruz; sc-166871, 1:200). All western blot were repeated in three biological replicates. See Supplementary Figure 1 for uncropped blots related to Fig. 2d.

### Genotyping by PCR

DNA was prepared by adding NaOH 50 mM (300 μl) to a freshly cut mouse tail (5 mm) and incubating it (100 °C, 2 h). Then, the digested tail was briefly vortexed and neutralized with Tris pH 7.5 (30 μl). Next, 3 μL of DNA was added to a master mix containing 2× Taq RED Master Mix (Apex, 5200300-1250) (5 μl) and forward and reverse primers (1 μM, 2 μl) and was submitted to PCR cycling (94 °C, 3 min, followed by 35 cycles (94 °C, 15 s; 60 °C, 45 s; and 72 °C, 45 s) and final elongation (72 °C, 5 min). The resulting PCR products were fractionated on 2% agarose gel.

### T cell proliferation assay

Following the manufacturer’s recommendations, CD3^+^ T cells were purified from the lymph nodes of *Nf1*^+/+^ and *Nf1*^+/−^ mice (age 3–6 months) using pan-T-cell isolation kit (Miltenyi Biotec, Auburn, CA). Purified CD3^+^ T cells (2 × 10^5^/well) were cultured for 2 days in 96-well plates (in triplicate) precoated with indicated doses of anti-CD3 Ab (eBioscience; cat. no. 16-0031-86) alone or anti-CD28 mAb (eBioscience; cat. no. 16-0281-85). After pulsing with ^3^H-thymidine (1 μCi/well [0.037 MBq/well]) for 20–22 h, cells were collected and evaluated for ^3^H radioactivity. For each experimental group, the T cell proliferation assay was performed in two pooled biological and three technical replicates.

###  DTH assay

*Nf1*^+/+^ (*n* = 3) and *Nf1*^+/−^ (*n* = 3) mice (age 3–6 months) were sensitized for DTH on day 0 by painting 2% oxazolone (Sigma) in acetone–olive oil (4:1 in volume) on shaved abdominal skin (sensitization). Mice were challenged on day 6 by painting 1% oxazolone and solvent control onto right and left ears, respectively (elicitation). Thereafter, DTH was assessed daily for 2 days by measuring ear thickness and calculating changes in ear swelling (thickness of right ear minus thickness of control left ear). For each experimental group, the T cell proliferation assay was performed in three biological replicates.

### Immunophenotyping of draining lymph node

For proliferation, draining lymph node cells (2 × 10^5^/well) of *Nf1* ^+/−^ and *Nf1* ^+/−^ mice were cultured without stimulation for 3 days and pulsed with ^3^H-thymidine (1 μCi/well [0.037 MBq/well]) for 20 h. For CD69 expression, LN cells were stained with FITC-anti-CD69 mAb (eBioscience; cat. no. 11-0691-85; 1:100) or FITC-isotypic control hamster IgG (BD Pharmingen; cat. no. 11114 C; 1:100) in the presence or absence of Ab directed at T cell (CD4 [eBioscience; cat. no. 17-0042-83; 1:100], CD8 [Lifetech; cat. no. A15836; 1:100], FoxP3 [eBioscience; cat. no. 12-57773-80; 1:100]), B-cell (B220 [eBioscience; cat. no. 11-0452-85; 1:100]), and pan hematopoietic (CD45 [eBioscience; cat. no. 25-0451-82; 1:100) cell surface marker and examined by FACS for surface expression of CD69 in each leukocyte subpopulation. Live cells were isolated from dead cells by staining with 7-aminoactinomycin D (Biolegend; 420403; 1:100). Analysis was performed in technical triplicates on three pooled biological replicates. See Supplementary Figure 2 for FACS gating strategies.

### Statistical analysis

A two-tailed *t-*test and Fisher exact test were applied as appropriate to evaluate statistical significance (*P* < 0.05). Variation was estimated using standard deviation with a 95% confidence level (Fig. 6). The distribution of the data in Fig. 5a is normal as judged by the kurtosis and skewness values.

## Electronic supplementary material

Supplementary Information

## Data Availability

All relevant data are available from the authors.
