# Supplementary material for: NF1 heterozygosity fosters de novo tumorigenesis but impairs malignant transformation
Source: Nat Commun. 2018 Nov 27;9:5014. doi: 10.1038/s41467-018-07452-y (PMC6258697; doi:10.1038/s41467-018-07452-y)
Supplement: Supplementary file 1 — Supplementary Information [file 41467_2018_7452_MOESM1_ESM.pdf]

Supplementary Figures and Tables for:

NF1 heterozygosity fosters de novo tumorigenesis but impairs malignant transformation

Jean-Philippe Brosseau[1#], Chung-Ping Liao[1#], Yong Wang[1], Vijay Ramani[1], Travis Vandergriff[1], Michelle Lee[1], Amisha Patel[1], Kiyoshi Ariizumi[1] and Lu Q Le[1,2,3,4,5]

[1] Department of Dermatology, [2] Neurofibromatosis Clinic, [3] Simmons Comprehensive Cancer Center, [4] Hamon Center for Regenerative Science and Medicine, University of Texas Southwestern Medical Center, Dallas, TX 75390, USA.

# These authors contribute equally

[5]Corresponding author contact information:

Lu Q Le, M.D., Ph.D.

Department of Dermatology
Simmons Comprehensive Cancer Center
University of Texas Southwestern Medical Center
5323 Harry Hines Blvd
Dallas, TX 75390-9069
Telephone: 214-648-5781
Fax: 214-648-5553
Email: lu.le@utsouthwestern.edu

Running Title: Antagonistic Role of *NF1* in Tumorigenesis

Keywords: Neurofibromatosis Type 1, NF1, Peripheral nerve sheath tumor, Schwann cells, Tumor microenvironment, benign tumor, two-step carcinogenesis, Tumor suppressor genes, Neurofibroma, Malignant peripheral nerve sheath tumor, MPNST.

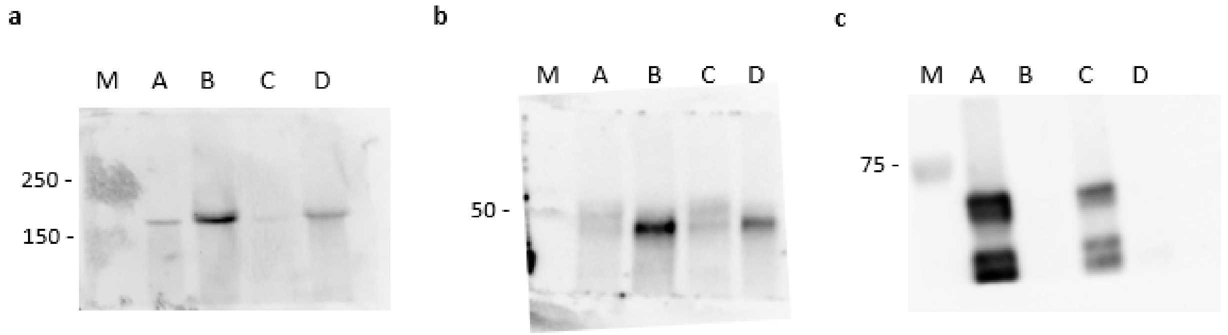

Supplementary Figure 1. Uncropped blots related to Figure 2d. **a:** anti-NF1 blot. **b:** anti-Lumican blot. **c:** anti-K1 blot. Lane M = marker; Lane A = epidermis from *Nf1*$^{f/f}$ mice; Lane B = dermis from *Nf1*$^{f/f}$ mice; Lane C = epidermis from *K14*Cre; *Nf1*$^{f/f}$ mice; Lane D = dermis from *K14*Cre; *Nf1*$^{f/f}$ mice.

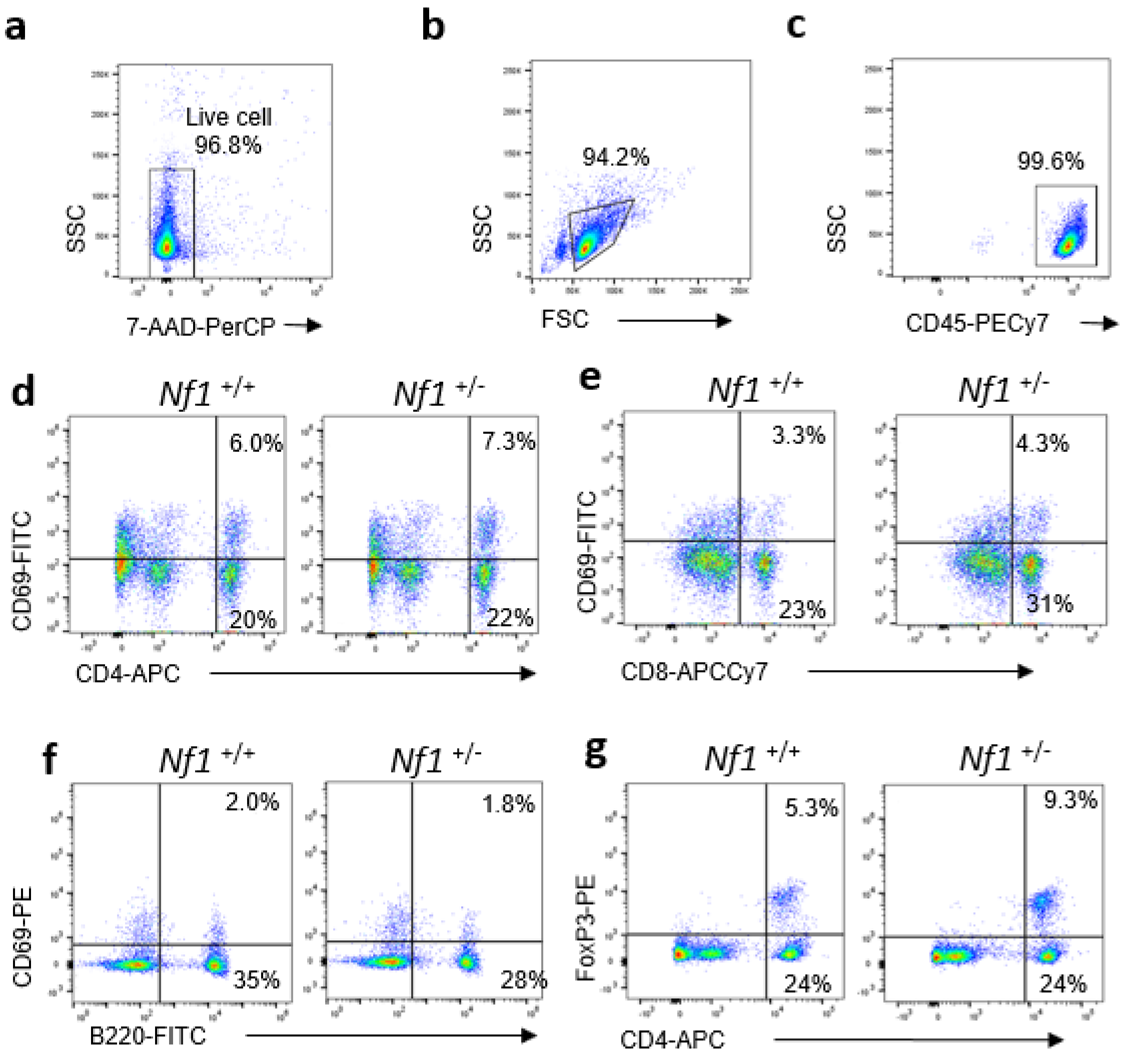

Supplementary Figure 2. FACS gating strategies related to Figure 6. **a:** Live cells gating by 7-aminoactinomycin D staining. **b:** Forward scatter (FSC) and side scatter (SSC) live cells gating. **c:** CD45 gating. **d-g:** CD69 and **d:** CD4 gating (related to Figure 6f); **e:** CD8 gating (related to Figure 6g); **f:** B220 gating (related to Figure 6h) and **g:** FOXP3 gating (related to Figure 6i).

Supplementary Table 1. The incidence of benign and malignant tumors commonly associated with NF1. The table summarizes the cell type; Neoplasm; incidence in both NF1 and nonNF1 patient and mean age at presentation for both benign and malignant NF1-related tumors; % of tumors mutated for *NF1*; prognosis in NF1 vs sporadic cases with a focus on the tumor commonly associated with NF1. **a** Less than 1% of the general population have multiple CALMs (more than 5) [1]. **b** From cancer.org; **c** Controversial. Maybe segmental and therefore associated with NF1. **d** From abta.org. **e** 10-25% of patient with sporadic GIST present at metastatic stage (cancer.gov). NR = Not reported; N/A = Not applicable.

Cell type	Benign / low metastatic potential lesions			Malignant / high metastatic potential tumors			
	**Neoplasm**	**mean age at presentation / Incidence in NF1 patient**	**mean age at presentation in nonNF1 patient / % of NF1 mutation**	**Tumor type**	**mean age at presentation / Incidence in NF1 patient**	**mean age at presentation in nonNF1 patient / % of NF1 mutation**	**Prognosis NF1 vs sporadic**
Melanocytes	Iris Hamartomas (Lisch nodules)	Childhood / >99% [2]	NR	Uveal melanoma	Rare [3]	58 [4-6] / Up to 60% [7]	N/A
	CALMs	Childhood / >99% [8]	Rare [a]	Cutaneous melanoma	4 cases reported [9,10]	63 [b] / Up to 30% [11,12]	N/A
Schwann cell lineage	Cutaneous neurofibroma	Around puberty / >99% [13]	Rare [14]	Malignant Peripheral nerve Sheath Tumor (MPNST)	NR	N/A	N/A
	Plexiform neurofibroma	Around puberty / Up to 27% [15,16]	Rare [c]	Malignant Peripheral nerve Sheath Tumor (MPNST)	40-50 [17] / Up to 10% [18]	50-60 [17] / Up to 72% [19]	Worse [18,20,21]
Glial cell	Low grade pilocytic astrocytomas	2 to 5 years Up to 15%	NR [22]	High grade Optic gliomas	Rare [23,24]	Childhood [25]	Better [25,26]
	Low grade non-optic glioma	Childhood or later in life / 5% [27]	childhood [28] / NR	High grade non-optic gliomas	Rare [29,30]	/ Up to 7% [31]	N/A
				Neuroblastoma	4 cases reported [32,33]	1-2 [b] / Up to 6%	N/A
				Glioblastoma	Rare [34]	59 [d] / Up to 23% [35]	NR
Interstitial cell of Cajal	Low grade GIST	50 [36] Up to 6% [37]	55-65 [36] / NR	Metastatic GIST	5 cases reported [38]	Frequent [e]	Better [39]
Endocrine cells of adrenal glands	Low grade Pheochromocytoma	42 [40] / Up to 6 % [40]	42 [41] / Up to 22% [42]	Metastatic pheochromocytoma	4 cases reported [43,44]	40-50 [45]	N/A
Myeloid cells	Juvenile Myelomonocytic leukemia (JMML) – chronic phase	Rare [46-48]	13 months [49] / 15-20% [50]	Juvenile Myelomonocytic leukemia (JMML) – blast crisis	NR	2	N/A
Breast epithelial cells	Carcinoma in situ	NR	NR	Breast cancer	Below 50 years Increased risk [51-55]	61[b] / Up to 3% [35]	Worse [54]

Supplementary Table 2. The incidence of sporadic benign and malignant tumors commonly associated with *NF1* mutation. The table summarizes the cell type; neoplasm; incidence in both NF1 and nonNF1 patient and means age at presentation for both benign and malignant tumors; % of tumors mutated for *NF1*; prognosis and/or risk factor in NF1 vs sporadic cases with a focus on the sporadic tumor typically associated with *NF1* mutation. **b** From cancer.org; **f** From ocrfa.org. NR = Not reported; N/A = Not applicable.

Cell type	Benign / low metastatic potential lesions			Malignant / high metastatic potential tumors			
	Neoplasms	Mean age at presentation / Incidence in NF1 patient	mean age at presentation in nonNF1 patient / % of NF1 mutation	Tumor type	Incidence in NF1 patient / mean age at presentation	mean age at presentation in nonNF1 patient / % of NF1 mutation	Prognosis NF1 vs sporadic
Melanocytes	NR	N/A	N/A	Desmoplastic melanoma	1 case reported [56]	66 [57] / / Up to 90% [58,59]	N/A
	Oral nevi	NR	N/A	Mucosal melanoma	NR	55-65 [60] / Up to 18% [61]	N/A
	NR	N/A	N/A	Conjunctival melanoma	3 cases reported [56]	60 / Up to 33% [62]	N/A
Myeloid cells	NR	NR	NR	AML	1 case reported [63]	67 [b] / Up to 7% [64,65]	N/A
Lymphoid cells	NR	NR	NR	T-ALL	Rare [66]	37 / 3% [67]	NR
Bladder epithelial cells	Papilloma and papillary hyperplasia	NR	N/A	Urinary tract transitional cell carcinoma	NR	61 [68] / Up to 7% [35,69]	N/A
Keratinocytes	papilloma	NR	40	Metastatic Cutaneous Squamous cell carcinoma	3 cases reported [70-72]	67 [73] / 10% [74]	N/A
Fallopian tube epithelial cells	Serous tubal intraepithelial lesion	NR	NR	Ovarian cancer	1 case reported [75]	63 [f] / Up to 34%	N/A
Lung epithelial cells	Carcinoma in situ	NR	NR	Lung carcinoma	Rare [76]	70 [b] / Up to 12% [35]	NR
Colon epithelial cells	Carcinoma in situ	NR	NR	Colorectal cancer	Rare [77-79]	70 [b] / Up to 6%	NR

Supplementary References

1 Boyd, K. P., Korf, B. R. & Theos, A. Neurofibromatosis type 1. *Journal of the American Academy of Dermatology* **61**, 1-14; quiz 15-16, doi:10.1016/j.jaad.2008.12.051 (2009).

2 Lubs, M. L., Bauer, M. S., Formas, M. E. & Djokic, B. Lisch nodules in neurofibromatosis type 1. *The New England journal of medicine* **324**, 1264-1266, doi:10.1056/NEJM199105023241807 (1991).

3 Honavar, S. G., Singh, A. D., Shields, C. L., Shields, J. A. & Eagle, R. C., Jr. Iris melanoma in a patient with neurofibromatosis. *Surv Ophthalmol* **45**, 231-236 (2000).

4 Shields, C. L. *et al.* Metastasis of uveal melanoma millimeter-by-millimeter in 8033 consecutive eyes. *Archives of ophthalmology* **127**, 989-998, doi:10.1001/archophthalmol.2009.208 (2009).

5 McLaughlin, C. C. *et al.* Incidence of noncutaneous melanomas in the U.S. *Cancer* **103**, 1000-1007, doi:10.1002/cncr.20866 (2005).

6 Shields, C. L., Kaliki, S., Furuta, M., Mashayekhi, A. & Shields, J. A. Clinical spectrum and prognosis of uveal melanoma based on age at presentation in 8,033 cases. *Retina* **32**, 1363-1372, doi:10.1097/IAE.0b013e31824d09a8 (2012).

7 Foster, W. J., Fuller, C. E., Perry, A. & Harbour, J. W. Status of the NF1 tumor suppressor locus in uveal melanoma. *Archives of ophthalmology* **121**, 1311-1315, doi:10.1001/archopht.121.9.1311 (2003).

8 Huson, S. M. a. K., B. in *Emery and Rimoin's Principles and Practice of Medical Genetics* Ch. 121, 1-45 (2013).

9 Gallino, G. *et al.* Association between cutaneous melanoma and neurofibromatosis type 1: analysis of three clinical cases and review of the literature. *Tumori* **86**, 70-74 (2000).

10 Salvi, P. F. *et al.* Cutaneous melanoma with neurofibromatosis type 1: rare association? A case report and review of the literature. *Ann Ital Chir* **75**, 91-95 (2004).

11 Krauthammer, M. *et al.* Exome sequencing identifies recurrent mutations in NF1 and RASopathy genes in sun-exposed melanomas. *Nat Genet* **47**, 996-1002, doi:10.1038/ng.3361 (2015).

12 Hodis, E. *et al.* A landscape of driver mutations in melanoma. *Cell* **150**, 251-263, doi:10.1016/j.cell.2012.06.024 (2012).

13 Widemann, B. C. Current status of sporadic and neurofibromatosis type 1-associated malignant peripheral nerve sheath tumors. *Curr Oncol Rep* **11**, 322-328 (2009).

14 Anastasaki, C., Dahiya, S. & Gutmann, D. H. KIR2DL5 mutation and loss underlies sporadic dermal neurofibroma pathogenesis and growth. *Oncotarget* **8**, 47574-47585, doi:10.18632/oncotarget.17736 (2017).

15 Waggoner, D. J., Towbin, J., Gottesman, G. & Gutmann, D. H. Clinic-based study of plexiform neurofibromas in neurofibromatosis 1. *American journal of medical genetics* **92**, 132-135 (2000).

16 Darrigo Jr, L. G., Geller, M., Bonalumi Filho, A. & Azulay, D. R. Prevalence of plexiform neurofibroma in children and adolescents with type I neurofibromatosis. *Jornal de pediatria* **83**, 571-573, doi:doi:10.2223/JPED.1718 (2007).

17 Patil, S. & Chamberlain, R. S. Neoplasms associated with germline and somatic NF1 gene mutations. *Oncologist* **17**, 101-116, doi:10.1634/theoncologist.2010-0181 (2012).
18 Ducatman, B. S., Scheithauer, B. W., Piepgras, D. G., Reiman, H. M. & Ilstrup, D. M. Malignant peripheral nerve sheath tumors. A clinicopathologic study of 120 cases. *Cancer* **57**, 2006-2021 (1986).
19 Lee, W. *et al.* PRC2 is recurrently inactivated through EED or SUZ12 loss in malignant peripheral nerve sheath tumors. *Nat Genet* **46**, 1227-1232, doi:10.1038/ng.3095 (2014).
20 Hagel, C. *et al.* Histopathology and clinical outcome of NF1-associated vs. sporadic malignant peripheral nerve sheath tumors. *Journal of neuro-oncology* **82**, 187-192, doi:10.1007/s11060-006-9266-2 (2007).
21 Valentin, T. *et al.* Management and prognosis of malignant peripheral nerve sheath tumors: The experience of the French Sarcoma Group (GSF-GETO). *European journal of cancer* **56**, 77-84, doi:10.1016/j.ejca.2015.12.015 (2016).
22 Dahiya, S., Yu, J., Kaul, A., Leonard, J. R. & Gutmann, D. H. Novel BRAF Alteration in a Sporadic Pilocytic Astrocytoma. *Case Rep Med* **2012**, 418672, doi:10.1155/2012/418672 (2012).
23 Rodriguez, F. J. *et al.* Gliomas in neurofibromatosis type 1: a clinicopathologic study of 100 patients. *J Neuropathol Exp Neurol* **67**, 240-249, doi:10.1097/NEN.0b013e318165eb75 (2008).
24 Rosenfeld, A., Listernick, R., Charrow, J. & Goldman, S. Neurofibromatosis type 1 and high-grade tumors of the central nervous system. *Childs Nerv Syst* **26**, 663-667, doi:10.1007/s00381-009-1024-2 (2010).
25 Robert-Boire, V., Rosca, L., Samson, Y., Ospina, L. H. & Perreault, S. Clinical Presentation and Outcome of Patients With Optic Pathway Glioma. *Pediatric neurology* **75**, 55-60, doi:10.1016/j.pediatrneurol.2017.06.019 (2017).
26 Helfferich, J. *et al.* Neurofibromatosis type 1 associated low grade gliomas: A comparison with sporadic low grade gliomas. *Critical reviews in oncology/hematology* **104**, 30-41, doi:10.1016/j.critrevonc.2016.05.008 (2016).
27 Sellmer, L. *et al.* Non-optic glioma in adults and children with neurofibromatosis 1. *Orphanet journal of rare diseases* **12**, 34, doi:10.1186/s13023-017-0588-2 (2017).
28 Sievert, A. J. & Fisher, M. J. Pediatric low-grade gliomas. *J Child Neurol* **24**, 1397-1408, doi:10.1177/0883073809342005 (2009).
29 Theeler, B. J. *et al.* Prolonged survival in adult neurofibromatosis type I patients with recurrent high-grade gliomas treated with bevacizumab. *J Neurol* **261**, 1559-1564, doi:10.1007/s00415-014-7292-0 (2014).
30 Byrne, S. *et al.* Clinical presentation and prognostic indicators in 100 adults and children with neurofibromatosis 1 associated non-optic pathway brain gliomas. *Journal of neuro-oncology* **133**, 609-614, doi:10.1007/s11060-017-2475-z (2017).
31 Wu, G. *et al.* The genomic landscape of diffuse intrinsic pontine glioma and pediatric non-brainstem high-grade glioma. *Nat Genet* **46**, 444-450, doi:10.1038/ng.2938 (2014).
32 Kushner, B. H., Hajdu, S. I. & Helson, L. Synchronous neuroblastoma and von Recklinghausen's disease: a review of the literature. *Journal of clinical oncology :*

official journal of the American Society of Clinical Oncology **3**, 117-120, doi:10.1200/JCO.1985.3.1.117 (1985).
33 Matsui, I. *et al.* Neurofibromatosis type 1 and childhood cancer. *Cancer* **72**, 2746-2754 (1993).
34 Huttner, A. J. *et al.* Clinicopathologic study of glioblastoma in children with neurofibromatosis type 1. *Pediatr Blood Cancer* **54**, 890-896, doi:10.1002/pbc.22462 (2010).
35 Kandoth, C. *et al.* Mutational landscape and significance across 12 major cancer types. *Nature* **502**, 333-339, doi:10.1038/nature12634 (2013).
36 Basile, U. *et al.* Gastrointestinal and retroperitoneal manifestations of type 1 neurofibromatosis. *J Gastrointest Surg* **14**, 186-194, doi:10.1007/s11605-009-0940-5 (2010).
37 Barahona-Garrido, J. *et al.* Association of GIST and somatostatinoma in a patient with type-1 neurofibromatosis: is there a common pathway? *The American journal of gastroenterology* **104**, 797-799, doi:10.1038/ajg.2008.133 (2009).
38 Miettinen, M., Fetsch, J. F., Sobin, L. H. & Lasota, J. Gastrointestinal stromal tumors in patients with neurofibromatosis 1: a clinicopathologic and molecular genetic study of 45 cases. *Am J Surg Pathol* **30**, 90-96 (2006).
39 Miettinen, M. & Lasota, J. Gastrointestinal stromal tumors: review on morphology, molecular pathology, prognosis, and differential diagnosis. *Arch Pathol Lab Med* **130**, 1466-1478, doi:10.1043/1543-2165(2006)130[1466:GSTROM]2.0.CO;2 (2006).
40 Walther, M. M., Herring, J., Enquist, E., Keiser, H. R. & Linehan, W. M. von Recklinghausen's disease and pheochromocytomas. *J Urol* **162**, 1582-1586 (1999).
41 Eisenhofer, G. *et al.* Age at diagnosis of pheochromocytoma differs according to catecholamine phenotype and tumor location. *The Journal of clinical endocrinology and metabolism* **96**, 375-384, doi:10.1210/jc.2010-1588 (2011).
42 Welander, J., Soderkvist, P. & Gimm, O. The NF1 gene: a frequent mutational target in sporadic pheochromocytomas and beyond. *Endocr Relat Cancer* **20**, C13-17, doi:10.1530/ERC-13-0046 (2013).
43 Gruber, L. M. *et al.* Pheochromocytoma and paraganglioma in patients with neurofibromatosis type 1. *Clin Endocrinol (Oxf)* **86**, 141-149, doi:10.1111/cen.13163 (2017).
44 Otoukesh, S. *et al.* Combination chemotherapy regimen in a patient with metastatic malignant pheochromocytoma and neurofibromatosis type 1. *Am J Case Rep* **15**, 123-127, doi:10.12659/AJCR.890181 (2014).
45 Bausch B., N. H. P. H. in *Neurofibromatosis Type 1* (ed Cooper D. Upadhyaya M.) 381-392 (Springer, Berlin, Heidelberg, 2012).
46 Gutmann, D. H., Gurney, J. G. & Shannon, K. M. Juvenile xanthogranuloma, neurofibromatosis 1, and juvenile chronic myeloid leukemia. *Arch Dermatol* **132**, 1390-1391 (1996).
47 Morier, P., Merot, Y., Paccaud, D., Beck, D. & Frenk, E. Juvenile chronic granulocytic leukemia, juvenile xanthogranulomas, and neurofibromatosis. Case report and review of the literature. *J Am Acad Dermatol* **22**, 962-965 (1990).

48 Shannon, K. M. *et al.* Loss of the normal NF1 allele from the bone marrow of children with type 1 neurofibromatosis and malignant myeloid disorders. *N Engl J Med* **330**, 597-601, doi:10.1056/NEJM199403033300903 (1994).
49 Side, L. E. *et al.* Mutations of the NF1 gene in children with juvenile myelomonocytic leukemia without clinical evidence of neurofibromatosis, type 1. *Blood* **92**, 267-272 (1998).
50 Flotho, C. *et al.* Genome-wide single-nucleotide polymorphism analysis in juvenile myelomonocytic leukemia identifies uniparental disomy surrounding the NF1 locus in cases associated with neurofibromatosis but not in cases with mutant RAS or PTPN11. *Oncogene* **26**, 5816-5821, doi:10.1038/sj.onc.1210361 (2007).
51 Evans, D. G. *et al.* Mortality in neurofibromatosis 1: in North West England: an assessment of actuarial survival in a region of the UK since 1989. *Eur J Hum Genet* **19**, 1187-1191, doi:10.1038/ejhg.2011.113 (2011).
52 Madanikia, S. A., Bergner, A., Ye, X. & Blakeley, J. O. Increased risk of breast cancer in women with NF1. *Am J Med Genet A* **158A**, 3056-3060, doi:10.1002/ajmg.a.35550 (2012).
53 Seminog, O. O. & Goldacre, M. J. Age-specific risk of breast cancer in women with neurofibromatosis type 1. *Br J Cancer* **112**, 1546-1548, doi:10.1038/bjc.2015.78 (2015).
54 Uusitalo, E. *et al.* Distinctive Cancer Associations in Patients With Neurofibromatosis Type 1. *J Clin Oncol* **34**, 1978-1986, doi:10.1200/JCO.2015.65.3576 (2016).
55 Wang, X. *et al.* Breast cancer and other neoplasms in women with neurofibromatosis type 1: a retrospective review of cases in the Detroit metropolitan area. *Am J Med Genet A* **158A**, 3061-3064, doi:10.1002/ajmg.a.35560 (2012).
56 Rubinstein, T. J., Plesec, T. P. & Singh, A. D. Desmoplastic melanoma of the eyelid and conjunctival melanoma in neurofibromatosis type 1: a clinical pathological correlation. *Surv Ophthalmol* **60**, 72-77, doi:10.1016/j.survophthal.2014.08.001 (2015).
57 Feng, Z., Wu, X., Chen, V., Velie, E. & Zhang, Z. Incidence and survival of desmoplastic melanoma in the United States, 1992-2007. *J Cutan Pathol* **38**, 616-624, doi:10.1111/j.1600-0560.2011.01704.x (2011).
58 Wiesner, T. *et al.* NF1 Mutations Are Common in Desmoplastic Melanoma. *The American journal of surgical pathology* **39**, 1357-1362, doi:10.1097/PAS.0000000000000451 (2015).
59 Shain, A. H. *et al.* The Genetic Evolution of Melanoma from Precursor Lesions. *The New England journal of medicine* **373**, 1926-1936, doi:10.1056/NEJMoa1502583 (2015).
60 Mihajlovic, M., Vlajkovic, S., Jovanovic, P. & Stefanovic, V. Primary mucosal melanomas: a comprehensive review. *Int J Clin Exp Pathol* **5**, 739-753 (2012).
61 Cosgarea, I. *et al.* Targeted next generation sequencing of mucosal melanomas identifies frequent NF1 and RAS mutations. *Oncotarget* **8**, 40683-40692, doi:10.18632/oncotarget.16542 (2017).

62 Scholz, S. L. *et al.* NF1 mutations in conjunctival melanoma. *Br J Cancer* **118**, 1243-1247, doi:10.1038/s41416-018-0046-5 (2018).
63 Sartor, C. *et al.* A case report of acute myeloid leukemia and neurofibromatosis 1. *Hematol Rep* **5**, 28-29, doi:10.4081/hr.2013.e8 (2013).
64 Boudry-Labis, E. *et al.* Neurofibromatosis-1 gene deletions and mutations in de novo adult acute myeloid leukemia. *American journal of hematology* **88**, 306-311, doi:10.1002/ajh.23403 (2013).
65 Parkin, B. *et al.* NF1 inactivation in adult acute myelogenous leukemia. *Clinical cancer research : an official journal of the American Association for Cancer Research* **16**, 4135-4147, doi:10.1158/1078-0432.CCR-09-2639 (2010).
66 Stiller, C. A., Chessells, J. M. & Fitchett, M. Neurofibromatosis and childhood leukaemia/lymphoma: a population-based UKCCSG study. *British journal of cancer* **70**, 969-972 (1994).
67 Kalender Atak, Z. *et al.* High accuracy mutation detection in leukemia on a selected panel of cancer genes. *PloS one* **7**, e38463, doi:10.1371/journal.pone.0038463 (2012).
68 Matalka, I., Bani-Hani, K., Shotar, A., Bani Hani, O. & Bani-Hani, I. Transitional cell carcinoma of the urinary bladder: a clinicopathological study. *Singapore Med J* **49**, 790-794 (2008).
69 Ross, J. S. *et al.* Advanced urothelial carcinoma: next-generation sequencing reveals diverse genomic alterations and targets of therapy. *Mod Pathol* **27**, 271-280, doi:10.1038/modpathol.2013.135 (2014).
70 Friedrich, R. E., Al-Dam, A. & Hagel, C. Squamous cell carcinoma of the sole of the foot in neurofibromatosis type 1. *Anticancer Res* **32**, 2165-2168 (2012).
71 Ishida, M. & Okabe, H. Cutaneous squamous cell carcinoma in a patient with neurofibromatosis type 1: A case report. *Oncol Lett* **6**, 878-880, doi:10.3892/ol.2013.1490 (2013).
72 Mancuso C, H. M., Austin C, ropper C, Hoffman C. A rare case of SCC in a Pediatric Patient with NF-1. *Journal of the American Osteopathic College of Dermatology* **37**, 44-45 (2017).
73 Vasconcelos, L., Melo, J. C., Miot, H. A., Marques, M. E. & Abbade, L. P. Invasive head and neck cutaneous squamous cell carcinoma: clinical and histopathological characteristics, frequency of local recurrence and metastasis. *An Bras Dermatol* **89**, 562-568 (2014).
74 Li, Y. Y. *et al.* Genomic analysis of metastatic cutaneous squamous cell carcinoma. *Clinical cancer research : an official journal of the American Association for Cancer Research* **21**, 1447-1456, doi:10.1158/1078-0432.CCR-14-1773 (2015).
75 Salud, A. *et al.* [Ovarian cancer in a female patient with von Recklinghausen's disease]. *Med Clin (Barc)* **96**, 138-140 (1991).
76 Gupta, K. B., Kumar, V., Tandon, S. & Gill, M. Primary carcinoma of the lung in von Recklinghausen neurofibromatosis. *Lung India* **26**, 130-132, doi:10.4103/0970-2113.56348 (2009).
77 Oh, S. H., Lee, J. H. & Namgung, H. A case of rectal cancer in a patient with neurofibromatosis type 1. *J Korean Soc Coloproctol* **28**, 170-173, doi:10.3393/jksc.2012.28.3.170 (2012).

78 Wood, J. J., Longman, R. J., Rooney, N., Loveday, E. J. & Roe, A. M. Colonic vascular anomalies and colon cancer in neurofibromatosis: report of a case. *Dis Colon Rectum* **51**, 360-362, doi:10.1007/s10350-007-9089-z (2008).
79 Kim, S. E., Heo, E. P., Yoon, T. J. & Kim, T. H. Segmentally distributed neurofibromatosis associated with adenocarcinoma of the colon. *The Journal of dermatology* **29**, 350-353 (2002).
